# Tuberculin skin testing and QuantiFERON™-TB Gold Plus positivity among household contacts in Vietnam

**DOI:** 10.5588/pha.23.0020

**Published:** 2023-09-21

**Authors:** A. L. Innes, S. T. Nguyen, V. Lebrun, T. T. H. Nguyen, T. P. Huynh, V. L. Quach, G. L. Hoang, T. B. Nguyen, T. B. P. Nguyen, H. M. Pham, A. Martinez, N. Dinh, V. L. Dinh, B. H. Nguyen, T. T. H. Truong, V. C. Nguyen, V. N. Nguyen, T. H. Mai

**Affiliations:** 1 FHI 360 Asia Pacific Regional Office, Bangkok, Thailand;; 2 University of California, San Francisco, CA, USA;; 3 FHI 360 Vietnam, Hanoi, Vietnam; 4 United States Agency for International Development Vietnam, Hanoi, Vietnam;; 5 FHI 360, Durham, NC, USA;; 6 Vietnam National Lung Hospital/National Tuberculosis Programme Hanoi, Vietnam

**Keywords:** latent tuberculosis infection, interferon-gamma release tests, tuberculin tests

## Abstract

**SETTING:**

TB infection (TBI) is diagnosed using the technique-dependent tuberculin skin test (TST) or costly, more accurate interferon-gamma release assays. The TST (⩾10 mm) threshold was indicated by previous research among household contacts in Vietnam, but routine implementation with a different tuberculin reagent showed unexpectedly low TST positivity.

**OBJECTIVE:**

TST (⩾5 mm and ⩾10 mm) results were compared to QuantiFERON™-TB Gold Plus (QFT) results in household contacts during community campaigns in 2020 and 2021.

**DESIGN:**

This was a cross-sectional multi-center implementation study.

**RESULTS:**

Among 1,330 household contacts in 2020, we found a TBI prevalence of 38.6% (QFT), similar to TST ⩾5 mm (37.4%) and higher than TST ⩾10 mm (13.1%). QFT+/TST+ was higher for TST ⩾5 mm (20.7%) than TST ⩾10 mm (9.4%). QFT was not discordant with TST ⩾5 mm (McNemar’s test = 0.6, *P* = 0.5) but was discordant with TST ⩾10 mm (McNemar’s test = 263.9, *P* < 0.01). Older age and Southern region increased odds for positive TST ⩾5 mm and QFT with weaker associations for TST ⩾10 mm. Agreement and discordance were similar in 2021 for 1,158 household contacts.

**CONCLUSION:**

Tuberculin reagents affect TST positivity rates. High TB burden countries should monitor reliability of TBI diagnosis, including tuberculin potency, cold chain, and TST technique to optimize eligibility for TB preventive treatment.

## Abstract (fr)

**CONTEXTE:**

L’infection tuberculeuse (TBI) est diagnostiquée à l’aide du test cutané à la tuberculine (TST), qui dépend de la technique, ou de tests de libération de l’interféron-gamma, coûteux et plus précis. Des recherches antérieures ont indiqué que le TST (⩾10 mm) est généralement utilisé pour diagnostiquer la TB parmi les contacts familiaux au Vietnam ; la mise en œuvre de routine avec un réactif de tuberculine différent a montré une faible positivité inattendue du TST.

**OBJECTIF:**

Les résultats du TST (⩾5 mm et ⩾10 mm) ont été comparés aux résultats de QuantiFERON™-TB Gold Plus (QFT) chez les contacts familiaux au cours des campagnes communautaires de 2020 et 2021.

**MÉTHODE:**

Il s’agissait d’une étude transversale multicentrique de mise en œuvre.

**RÉSULTATS:**

Parmi 1 330 contacts familiaux en 2020, nous avons trouvé une prévalence de TBI de 38,6% (QFT), similaire au TST ⩾5 mm (37,4%) et plus élevée que le TST ⩾10 mm (13,1%). Le QFT+/TST+ était plus élevé pour le TST ⩾5 mm (20,7%) que pour le TST ⩾10 mm (9,4%). Le QFT n’était pas discordant avec le TST ≥5 mm (test de McNemar = 0,6 ; *P* = 0,5) mais était discordant avec le TST ⩾10 mm (test de McNemar = 263,9 ; *P* < 0,01). L’âge avancé et la région méridionale augmentaient les probabilités d’un TST positif ⩾5 mm et d’un QFT, avec des associations plus faibles pour un TST ⩾10 mm. La concordance et la discordance étaient similaires en 2021 pour 1 158 contacts familiaux.

**CONCLUSION:**

Les réactifs de tuberculine affectent les taux de positivité des TST. Les pays à forte charge de TB doivent surveiller la fiabilité du diagnostic de TBI, y compris la puissance de la tuberculine, la chaîne du froid et la technique du TST afin d’optimiser l’éligibilité au traitement préventif de la TB.

Approximately one-fourth of the world’s population has TB infection (TBI),^1^ of whom an estimated 5–10% are at risk for progression to TB disease.^2^ The highest risk for progression is within the first 2 years of infection, especially among children younger than 5 years old and those with immune suppression, including people living with HIV (PLWH), silicosis and kidney disease.^3^ As a reservoir of TB disease, TBI is a major barrier to ending TB.^4^,^5^ In high TB burden countries, TB preventive treatment (TPT) was previously prioritized in PLWH and child contacts (less than 5 years) of bacteriologically confirmed pulmonary TB disease. In these groups, TPT required TB disease exclusion but not TBI diagnosis.^6^ The WHO’s updated consolidated guidelines for latent TBI^2^ recommends that TPT may be given to HIV-negative household contacts of all ages without TB disease. High TB burden countries must determine whom to treat: excluding TB disease alone expands eligibility for TPT, while diagnosing TBI is complicated by suboptimal testing methods. The tuberculin skin test (TST) remains the TBI diagnostic standard of choice in high TB burden settings,^5^ where it is less specific due to cross-reactivity with antigens from recent bacilli Calmette-Guérin (BCG) vaccination^7^ and environmental non-tuberculous mycobacteria. TST administration and interpretation are operator-dependent,^8^ and results may vary according to the purified protein derivative (PPD) tuberculin used.^9^ Global shortage of tuberculin and growing numbers of locally manufactured tuberculin reagents are a challenge to standardization and quality assurance.^10^ Interferon-gamma release assays (IGRAs) are more specific than TST and better at predicting progression to TB disease,^11^,^12^ but are expensive and require laboratory capacity. There is no gold standard to diagnose TBI since methods only detect immune response to TB antigens.^2^,^13^ To determine clinical sensitivity, TBI diagnostic tests are conducted among individuals with bacteriologically confirmed TB disease and compared against WHO-recommended IGRA reference tests.^13^,^14^

Vietnam is eleventh among 30 countries with the highest TB burden;^15^ an estimated 169,000 individuals developed TB disease in 2021.^16^ Ending TB requires reducing TB incidence by 90%, and modelling shows that both TB disease and TBI interventions are required to achieve this goal. The Vietnam National TB Programme (NTP) is implementing active case-finding (ACF) community campaigns to detect TB disease and link individuals to treatment. These campaigns initially prioritized TB disease and, since 2020, integrated TBI for household contacts of all ages.

Both TST and IGRA (QuantiFERON®-TB Gold Plus [QFT]; Qiagen, Hilden, Germany) are approved in Vietnam to diagnose TBI, but TST is preferred for routine implementation. The TST ⩾10 mm threshold was based on Vietnam’s first national tuberculin survey in child contacts (6–14 years) of bacteriologically confirmed index pulmonary TB, which was nested within the 2007 TB prevalence survey;^17^ TBI prevalence (TST ⩾ 10 mm) was 16.7% using tuberculin RT23/Tween 80 (Statens Serum Institute, Copenhagen, Denmark). Another study in Vietnam showed 25.8% TST ⩾10 mm positivity among contacts of new TB patients and 40.8% among contacts of multidrug-resistant TB (MDR-TB) patients.^18^ In 2020, the Vietnam NTP implemented community campaigns using a different ­tuberculin reagent (BulBio, BB-NCIPD Ltd, Sofia, Bulgaria) than the prevalence survey. TBI prevalence (TST ⩾10 mm) among household contacts was lower than expected, prompting QFT testing in a subset of household contacts to evaluate agreement with TST and determine the best TST threshold (⩾5 mm or ⩾10 mm) for TBI. TST and QFT were administered in a different subset of household contacts in 2021.

## METHODS

### Study population

This was a cross-sectional, multi-center study implemented under routine program conditions during community campaigns screening for TB disease and TBI (2020 and 2021). Campaign sites were conveniently selected in nine districts of five provinces that reported approximately 20% of TB notifications in Vietnam and represented the Northern, Central and Southern regions, which vary in terms of TB prevalence.^19^,^20^ District campaign sites in each province differed by year, with the strategy to cover all districts over the implementation period. Campaign size was estimated from districts’ index patients with pulmonary TB disease and their household contacts. The QFT subset was conveniently sampled among household contacts in Southern and Northern/Central regions; sample size was determined by capacity and budget for QFT testing.

Index patients were adults diagnosed with pulmonary drug-susceptible TB disease (clinically or bacteriologically confirmed) and notified in NTP registers, within 2 years of each campaign. Home visits identified household contacts, defined as those who 1) slept in the same house with a pulmonary TB patient for at least one night/week for 3 months before diagnosis, or 2) stayed in the same house with a pulmonary TB patient for at least 1 h/day over 5 consecutive days/week for 3 months before TB disease diagnosis. Campaign exclusion criteria were COVID-19 positivity or no verbal consent. ACF campaigns screened other vulnerable populations (elderly, smokers, diabetics) for TB disease, but TBI testing prioritized household contacts.

### Testing for TBI diagnosis

District NTP staff administered TST for household contacts excluding those younger than 5 years or pregnant. Using the Mantoux method, 0.1 mL of 5 tuberculin units of BulBio tuberculin was injected intradermally into the forearm. Transverse diameter induration at the injection site was measured using pen and palpation after 48–72 h. QFT was conducted by or under the supervision of Tekmax Co, Hanoi, Vietnam), the main vendor in Vietnam performing IGRA. Venous blood (4 mL) was collected for QFT analysis immediately before TST injection and processed according to the manufacturer’s instructions, with ⩾0.35 international units (IU)/mL as the positivity cut-off. TB disease was excluded among household contacts using chest X-ray and sputum Xpert^®^ MTB/RIF or Xpert^®^ Ultra (Cepheid, Sunnyvale, California, USA) testing.

### Ethics review

Ethics approval was not required since community campaigns were routinely implemented to diagnose and treat TB disease and TBI; QFT was conducted as quality assurance to evaluate TST. Participants gave verbal consent.

### Statistical analysis plan

Comparative analyses were used to evaluate the positivity rate, agreement, and discordance of TST (⩾5 mm and ⩾10 mm) and QFT. Raw agreement was calculated as a proportion of the total TST ⩾5 mm/⩾10 mm and QFT results for which there was positive agreement (QFT+/TST+), negative agreement (QFT–/TST–), and discordance (QFT+/TST– and QFT–/TST+). Agreement between TST ⩾5 mm/⩾10 mm and QFT was measured using Cohen’s κ. Discordance was measured using McNemar’s test to determine if TST ⩾ 5 mm and TST ⩾ 10 mm had the same marginal probabilities as QFT. χ^2^ tests of independence for TST ⩾5 mm/⩾10 mm and QFT positivity by age group, sex and region were conducted. Univariable and multivariable logistic regression modelling of TST ⩾5 mm/⩾10 mm and QFT positivity included all available variables (age group, sex, region and symptoms) as predictors. Logistic regression reference groups were female sex; district with the highest participants each year; and age group with lowest odds for test positivity from univariable analysis (10 to less than 20). All analyses were conducted in Stata v17 (Stata, College Station, TX, USA). Data from 2020 and 2021 were analyzed separately due to different conditions in 2021, when campaigns with full-body personal protective equipment for health staff immediately followed COVID-19 vaccinations.

## RESULTS

**FIGURE 1:**
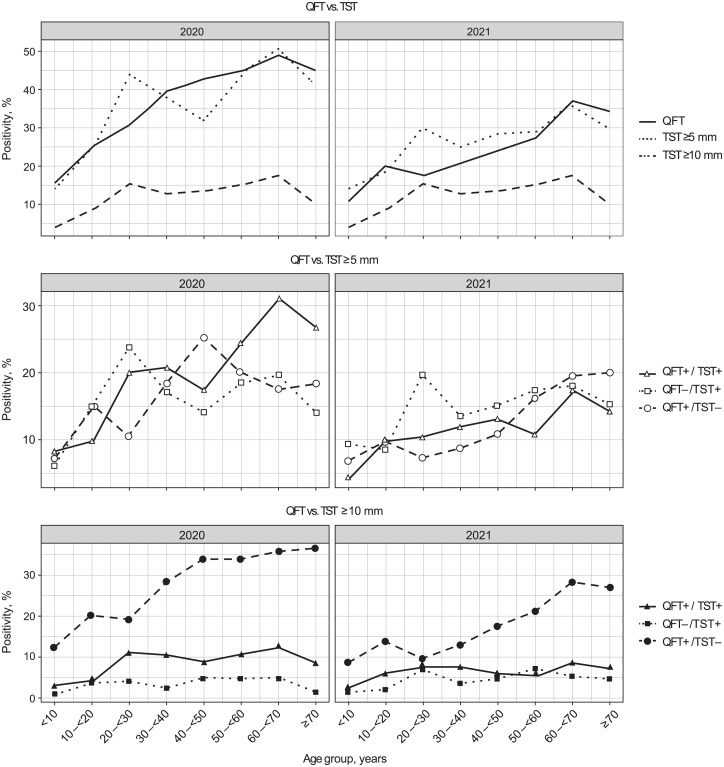
Age-specific prevalence for QFT, TST ⩾5 mm and TST ⩾10 mm positivity among household contacts in 2020 and 2021. Upper: Positivity rates for QFT (solid line), TST ⩾5 mm (dotted line) and TST ⩾10 mm (dashed line) by age groups in 2020 (left) and 2021 (right). Middle: Rates of concordant positivity for QFT and TST ⩾5 mm (open triangle); TST ⩾5 mm-only positivity (open square); and QFT-only positivity (open circle) by age groups in 2020 and 2021. All TST ⩾5 mm curves show an increase in TST positivity with increasing age. Lower: Rates for concordant positivity of QFT and TST ⩾10 mm (closed triangle); TST ⩾10 mm-only positivity (closed square); and QFT-only positivity (closed circle) by age groups. TST ⩾10 mm-only positivity (QFT–/TST+, closed squares) is low and does not vary with age, in contrast to TST ⩾5 mm. Corresponding with Middle and Lower panels, Supplementary Table S2 shows results from pairwise comparisons of age-specific positivity rates for QFT, TST ⩾5 mm and TST ⩾10 mm. QFT = QuantiFERON-TB Gold Plus; TST = tuberculin skin testing; + = positive; – = negative.

**FIGURE 2:**
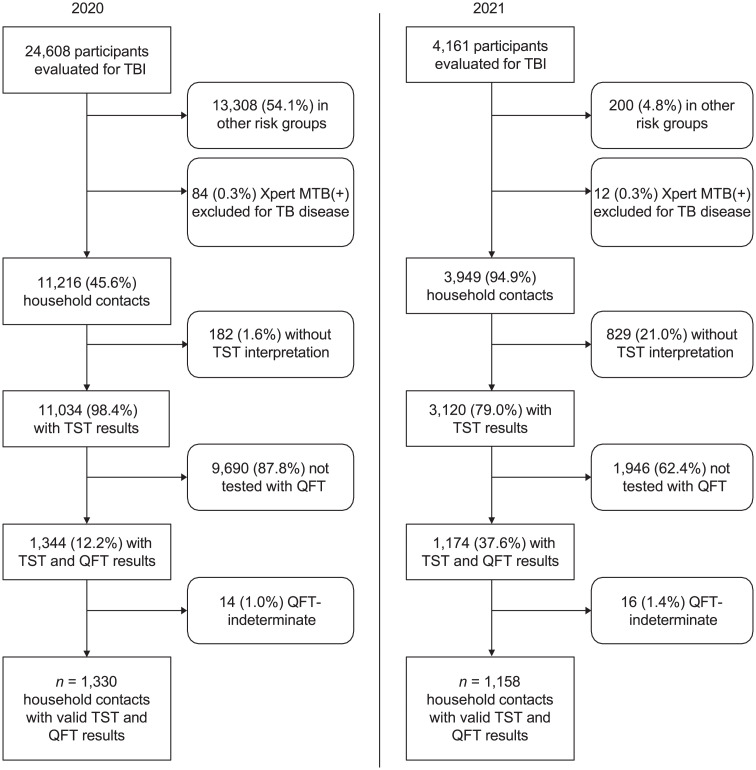
Participants in TB ACF campaigns evaluated with QFT and TST in 2020 and 2021. A subset of household contacts from ACF campaigns was evaluated for TBI with both TST and QFT. ACF participants were screened for TBI and TB disease; those with Xpert-confirmed pulmonary TB disease or with QFT-indeterminate results were excluded from analyses. TBI = TB infection; + = positive; TST = tuberculin skin testing; QFT = QuantiFERON-TB Gold Plus; ACF = active case-finding.

From March to December 2020, QFT and TST were performed on 1,330 household contacts among 24,608 campaign participants after excluding TB disease (Figure 2). Household contacts had a mean age of 41.7 years (standard deviation [SD] 19.4); 63.5% were female and 76.9% Southern (Table 1). Quantitative QFT results showed a low median, wide distribution for positive QFT, and clustering close to the threshold (Table 2, Supplementary Figure S1). Positive test prevalence was 38.6%, 37.4%, and 13.1% for QFT, TST ⩾ 5 mm, and TST ⩾ 10 mm, respectively (Table 3). Overall agreement with QFT was similar for both thresholds, but TST ⩾ 5 mm produced higher concordant positivity (20.7%) and similar discordance in both directions (QFT+/TST– positivity: 18.0%; QFT–/TST+ positivity: 16.8%). TST ⩾ 10 mm produced higher concordant negativity (57.7%) and higher QFT+/TST– positivity (29.2%) than QFT–/TST+ (3.7%) (odds ratio [OR] 7.9, 95% confidence interval [CI] 5.9–10.9; Table 4). We found slight to fair agreement between TST ⩾ 5 mm/TST ⩾ 10 mm and QFT (TST ⩾ 5 mm: κ = 0.3; TST ⩾ 10 mm: κ = 0.2) (Table 4). Marginal probabilities for TST ⩾ 5 mm and QFT were not statistically different (McNemar’s test = 0.6, *P* = 0.5), while they were different, or discordant, for TST ⩾ 10 mm and QFT (McNemar’s test = 263.9, *P* < 0.01). Age group and region, but not sex, were associated with TST ⩾ 5 mm, TST ⩾ 10 mm and QFT positivity (Supplementary Table S1) in multivariable regression modelling. Age groups older than 30 years had higher odds for positive QFT and TST ⩾ 5 mm, while only those from 60 to under 70 years had increased odds for positive TST ⩾ 10 mm (adjusted OR [aOR] 2.4, 95% CI 1.2–5.0). The South had higher odds for positive QFT (aOR 2.0, 95% CI 1.5–2.6) and TST ⩾ 5 mm (aOR 4.1, 95% CI 2.9–5.8) than the North/Central Regions. TBI prevalence increased with age for both QFT and TST ⩾ 5 mm but not for TST ⩾ 10 mm (Figure 1, Upper). Age-specific QFT-only positivity (QFT+/TST–) for TST ⩾ 10 mm was higher than TST ⩾ 10 mm-only positivity (QFT–/TST+) (Figure 1, Lower; Supplementary Table S2, Lower).

**TABLE 1:** Baseline characteristics of household contacts evaluated with QFT and TST in 2020 and 2021

	2020 (*n* = 1,330) *n* (%)	2021 (*n* = 1,158) *n* (%)
Age, years*	(*n* = 1,327)	(*n* = 1,157)
0–<10	98 (7.4)	118 (10.2)
10–<20	133 (10.0)	151 (13.1)
20–<30	125 (9.4)	96 (8.3)
30–<40	217 (16.4)	185 (16.0)
40–<50	218 (16.4)	146 (12.6)
50–<60	262 (19.7)	222 (19.2)
60–<70	203 (15.3)	154 (13.3)
⩾70	71 (5.4)	85 (7.3)
Sex*	(*n* = 1,330)	(*n* = 1,124)
Female	844 (63.5)	709 (63.1)
Male	486 (36.5)	415 (36.9)
District		
Tien Hai (North)	307 (23.1)	
Trang Bang (South)	371 (27.9)	
Ninh Kieu (South)	349 (26.2)	
Bien Hoa (South)	303 (22.8)	
Dong Hung (North)		115 (9.9)
Yen Thanh (Central)		228 (19.7)
Thanh Binh (South)		215 (18.6)
Chau Thanh (South)		466 (40.2)
Thoi Lai (South)		134 (11.6)
Region		
North	307 (23.1)	115 (9.9)
Central	0 (0)	228 (19.7)
South	1,023 (76.9)	815 (70.4)
TST induration, mm		
<5	832 (62.6)	853 (73.7)
5–9	324 (24.4)	176 (15.2)
10–14	139 (10.5)	101 (8.7)
⩾15	35 (2.6)	28 (2.4)
QFT, IU/mL^†^	(*n* = 1,344)	(*n* = 1,174)
Negative	816 (60.7)	879 (74.9)
Positive	514 (38.2)	279 (23.8)
Indeterminate	14 (1.0)	16 (1.4)

*Some data are missing for age and sex.

†QFT-indeterminate results were excluded from all analyses.

QFT = QuantiFERON-TB Gold Plus; IU = international units; TST = tuberculin skin testing.

**TABLE 2:** QFT results for household contacts in 2020 and 2021

	2020 (*n* = 1,344)		2021 (*n* = 1,174)	
QFT, IU/mL*	TB1-Nil Median [IQR]	TB2-Nil Median [IQR]	TB1-Nil Median [IQR]	TB2-Nil Median [IQR]
Negative	0.00 [–0.02 to 0.04]	0.02 [–0.01 to 0.08]	0.00 [–0.02 to 0.03]	0.01 [–0.01 to 0.06]
Positive	1.54 [0.62 to 3.64]	1.57 [0.71 to 3.76]	1.07 [0.49 to 2.72]	1.16 [0.58 to 2.79]
Indeterminate	0.01 [–0.01 to 0.03]	0.01 [0.00 to 0.12]	0.01 [–0.03 to 0.13]	0.08 [0.01 to 0.28]

*QFT-indeterminate results were excluded from all analyses.

QFT = QuantiFERON-TB Gold Plus; IU = international units; IQR = interquartile range.

**TABLE 3:** Positive prevalence for QFT, TST ⩾5 mm and TST ⩾10 mm among household contacts by region, sex and age groups in 2020 and 2021

	2020				2021			
	*n*	QFT *n* (%)	TST ⩾5 mm *n* (%)	TST ⩾10 mm *n* (%)	*n*	QFT *n* (%)	TST ⩾5 mm *n* (%)	TST ⩾10 mm *n* (%)
Overall	1,330	514 (38.6)	498 (37.4)	174 (13.1)	1,158	279 (24.1)	305 (26.3)	129 (11.1)
Region								
South	1,023	428 (41.8)	448 (43.8)	141 (13.8)	815	213 (26.1)	218 (26.7)	95 (11.7)
Other*	307	86 (28.0)	50 (16.3)	33 (10.7)	343	66 (19.2)	87 (25.4)	34 (9.9)
Sex								
Female	844	325 (38.5)	308 (36.5)	108 (12.8)	709	173 (24.4)	168 (23.7)	74 (10.4)
Male	486	189 (38.9)	190 (39.1)	66 (13.6)	415	97 (23.4)	127 (30.6)	52 (12.5)
Age group, years								
0–<10	98	15 (15.3)	14 (14.3)	4 (4.1)	118	13 (11.0)	16 (13.6)	5 (4.2)
10–<20	133	33 (24.8)	33 (24.8)	11 (8.3)	151	30 (19.9)	28 (18.5)	12 (7.9)
20–<30	125	38 (30.4)	55 (44.0)	19 (15.2)	96	17 (17.7)	29 (30.2)	15 (15.6)
30–<40	217	85 (39.2)	82 (37.8)	28 (12.9)	185	38 (20.5)	47 (25.4)	21 (11.4)
40–<50	218	93 (42.7)	69 (31.7)	29 (13.3)	146	35 (24.0)	41 (28.1)	16 (11.0)
50–<60	262	117 (44.7)	113 (43.1)	40 (15.3)	222	60 (27.0)	63 (28.4)	29 (13.1)
60–<70	203	99 (48.8)	103 (50.7)	36 (17.7)	154	57 (37.0)	55 (35.7)	21 (13.6)
⩾70	71	32 (45.1)	29 (40.8)	7 (9.9)	85	29 (34.1)	25 (29.4)	10 (11.8)

*North (2020); North and Central (2021).

QFT = QuantiFERON-TB Gold Plus; TST = tuberculin skin testing.

**TABLE 4:** Agreement and discordance for QFT vs. TST ⩾5 mm and TST ⩾10 mm among household contacts in 2020 and 2021*

	QFT+/TST+	QFT–/TST–	QFT+/TST–	QFT–/TST+	Cohen’s κ agreement			McNemar’s test (probability)
	*n* (%)	*n* (%)	*n* (%)	*n* (%)	Total	Expected	κ (SE)	
2020 (*n* = 1,330)								
TST ⩾5 mm	275 (20.7)	593 (44.6)	239 (18.0)	223 (16.8)	65.3	52.9	0.3 (0.03)	0.6 (0.5)
TST ⩾10 mm	125 (9.4)	767 (57.7)	389 (29.2)	49 (3.7)	67.1	58.4	0.2 (0.02)	263.9 (<0.01)
2021 (*n* = 1,158)								
TST ⩾5 mm	134 (11.6)	708 (61.1)	145 (12.5)	171 (14.8)	72.7	62.3	0.3 (0.03)	2.1 (0.1)
TST ⩾10 mm	75 (6.5)	825 (71.2)	204 (17.6)	54 (4.7)	77.7	70.1	0.3 (0.03)	87.2 (<0.01)

*Raw concordant agreement (QFT+/TST+; QFT–/TST–) and discordance (QFT+/TST–; QFT–/TST+) are shown for both TST thresholds, comparing both thresholds against QFT. Cohen’s κ measures agreement and McNemar’s test measures discordance. Using the exact McNemar’s test conditioned on discordant pairs, in 2020, the TST ⩾10 mm QFT+/TST– percentage was statistically different from the QFT–/TST+ percentage (29.2% vs. 3.7%; OR 7.9, 95% CI 5.9–10.9). In 2021, TST ⩾10 mm QFT+/TST– was statistically different from QFT–/TST+ (17.6% vs. 4.7%; OR 3.8, 95% CI 2.8–5.2).

QFT = QuantiFERON-TB Gold Plus; TST = tuberculin skin testing; + = positive; – = negative; SE = standard error; OR = odds ratio, CI = confidence interval.

From July to October 2021, movement in Vietnam restricted due to COVID-19, preventing community campaigns that resumed after COVID-19 vaccinations. From October to December 2021, TST and QFT were performed in 1,158 household contacts from five districts in the Northern, Central and Southern Regions (Figure 2, Tables 1 and 2); the mean age was 40.3 years (SD 21.2); 63.1% were female, and 70.4% Southern. TBI prevalence was lower overall in 2021 than in 2020 but similar for QFT (24.1%) and TST ⩾ 5 mm (26.3%) compared to TST ⩾ 10 mm (11.1%) (Table 3). Agreement and discordance between QFT and TST ⩾ 5 mm/TST ⩾ 10 mm were similar to 2020 (Table 4). Associations for age group and region with test positivity were weaker overall in 2021 (Supplementary Table S1): older age had higher odds for positive QFT and TST ⩾ 5 mm and the South had higher odds for positive QFT (aOR 1.5, 95% CI 1.1–2.1). Males had higher odds for positive TST ⩾ 5 mm (aOR 1.6, 95% CI 1.2–2.1) in 2021, which was not found in 2020. Age, sex and region were not associated with positive TST ⩾ 10 mm in 2021.

## DISCUSSION

Vietnam’s End TB Strategy prioritizes scaling up TPT among all household contacts, but lower than expected TST positivity rates in routine implementation limited contacts’ eligibility for TPT. QFT comparison showed higher positive agreement and lower discordance with TST ⩾ 5 mm than ⩾10 mm thresholds. Lower tuberculin potency was identified as the likely explanation for these programmatic results.

Patterns of regional TBI prevalence in our study are consistent with TB disease prevalence in Vietnam, which is highest in the Southern and lower in the Northern and Central Regions.^19^,^20^ Our results show that among household contacts, living in the Southern Region and older age are stronger predictors of TBI than sex. TBI prevalence normally increases with age for adult household contacts and general populations in high TB burden settings due to increased risk for TB exposure over time, while TBI prevalence declines in the >70 years from waning immunity. In our population, TST ⩾ 5 mm and QFT positivity increased, as expected with age, consistent with studies in India^21^ and China,^22^ while TST ⩾ 10 mm positivity did not vary with age. Lower potency tuberculin could result in a delayed hypersensitivity response robust enough for induration ⩾5 mm, but not ⩾10 mm.

Being male did not increase the odds for TBI positivity, except for TST ⩾5 mm in 2021. This suggests that sex is not as strong a risk factor for TBI as it is for TB disease, for which the male:female ratio is approximately 4:1 in Vietnam. A previous study in Vietnam’s Southern province of Ca Mau showed that more than one third of the general adult population had TBI, which was higher in males, but the sex difference between males and females was even greater for TB disease notification.^23^ TBI risk may be similar for male or female household contacts, while vulnerability for TB disease is significantly higher for males due to factors that promote TB progression or transmission, such as smoking, alcohol use, or exposure in work and social settings. Studies in other countries have shown no or minimal difference for TBI prevalence by sex among groups with higher TB disease prevalence in males.^21^,^24^

TST and IGRA (QFT-Plus or QuantiFERON-TB^® ^Gold In-Tube [QFT-GIT]) positivity rates vary in high-burden countries.^21^,^22^,^25^ One study in India^26^ showed a TBI prevalence of 71% among household contacts tested with both QFT-GIT and TST (⩾5 mm and ⩾10 mm). Neither QFT-GIT nor TST at either threshold predicted incident TB disease after 24 months. Various tuberculin reagents manufactured in India have yielded different TBI prevalence rates among household contacts.^21^,^27^ A study of TB outbreaks in Chinese schools^28^ calibrated the TST threshold against QFT-GIT, which was lower for contacts in the same classroom as the index (TST ⩾ 9 mm) than contacts in other classrooms (TST ⩾ 10.5 mm); China’s guideline recommends TST ⩾15 mm.^28^

In vitro and animal studies^9^ or direct in vivo comparison^29^ with tuberculin standards evaluate potency for tuberculin reagents. Variability in the molecular composition of reagents limits root cause identification when potency differs. In Vietnam, BulBio tuberculin’s potency was identified as the likely explanation for lower TST positivity in routine implementation, after evaluating cold chain and addressing TST administration skills. In the Netherlands, BulBio tuberculin produced larger indurations than RT23 and Tubersol (5TE),^30^ suggesting variable BulBio potency. For over 50 years, tuberculin potency was standardized using one product (PPD-Standard), but multiple standards are now used globally to evaluate tuberculin potency.^10^ Changes in tuberculin reagent can affect TST results.^31^ To mitigate challenges for TPT eligibility, high-burden countries may choose to give TPT to household contacts of all ages after excluding TB disease and assessing clinical risk if TBI diagnostic testing is unavailable or unreliable.^2^,^32^ Countries using TST to determine eligibility for TPT should monitor tuberculin reagent, potency, and cold chain, as well as TST quality assurance.

Despite training and ongoing monitoring, inter- and intra-operator variability for TST administration remains a limitation of our results. Our study findings on TST ⩾ 10 mm positivity differed from those using BulBio tuberculin in a Vietnamese study,^18^ which was conducted in one province vs. our programmatic implementation in five provinces. We did not compare TST induration for BulBio head-to-head with tuberculin standards (e.g., RT23). Campaigns involved routine programming without controlling for clinical or demographic variables, with no data collected on BCG vaccination or scar. We evaluated TBI testing in household contacts, in whom clinical sensitivity of TST and the QFT reference cannot be calculated, as would be possible with confirmed TB disease. Our TST/QFT comparative analyses were also subject to potential QFT variability in field applications. Finally, TBI prevalence overall was lower in 2021 for both QFT and TST than in 2020. Explanations include the effect of the 2021 COVID-19 lockdowns on campaigns, the complexity of COVID-safe campaigns impacting testing quality by both methods, and recent COVID-19 vaccination. Inactive vaccines should not impact the immune response for TST or QFT, but data on this are limited.

New TBI diagnostic methods are needed for the programmatic expansion of TPT.^33^ TB antigen-based skin testing is approved by the WHO to diagnose TBI,^34^ with similar specificity to IGRA, similar sensitivity to TST and IGRA, and one (5 mm) threshold.^35^ Near point-of-care QIAreach QuantiFERON^®^-TB (Qiagen; Carnegie, VIC, Australia) is a less expensive, simpler alternative to QFT.^14^ Programmatic testing for TBI in high-burden countries requires accurate, quality-assured methods that can be decentralized and are affordable at scale. High TB burden countries expanding TPT to all household contacts should monitor TBI diagnostic performance, including quality assurance for tuberculin and TST. TPT strategies should align with availability and reliability of testing to exclude TB disease and diagnose TBI, guided by local epidemiology for TB disease and TBI.

## References

[1] Houben RMGJ, Dodd PJ (2016). The global burden of latent tuberculosis infection: a re-estimation using mathematical modelling. PLOS Med.

[2] World Health Organization (2020). WHO consolidated guidelines on tuberculosis. Module 1: prevention - tuberculosis preventive treatment, 2020.

[3] Getahun H (2015). Latent *Mycobacterium tuberculosis* infection. N Engl J Med.

[4] Harries AD (2020). The growing importance of tuberculosis preventive therapy and how research and innovation can enhance its implementation on the ground. Trop Med Infect Dis.

[5] Paton NI (2019). Diagnosis and management of latent tuberculosis infection in Asia: Review of current status and challenges. Int J Infect Dis.

[6] World Health Organization (2015). Guidelines on the management of latent tuberculosis infection.

[7] Zwerling A (2011). The BCG World Atlas: a database of global BCG vaccination policies and practices. PLoS Med.

[8] Faria N, Reis R (2022). Screening for TB infection: the operator´s impact. Int J Tuberc Lung Dis.

[9] Yang H, Kruh-Garcia NA, Dobos KM (2012). Purified protein derivatives of tuberculin–past, present, and future. FEMS Immunol Med Microbiol.

[10] Duthie MS, Reed SG (2021). Skin tests for the detection of Mycobacterial infections: achievements, current perspectives, and implications for other diseases. Appl Microbiol Biotechnol.

[11] Ho CS (2022). Comparison of three tests for latent tuberculosis infection in high-risk people in the USA: an observational cohort study. Lancet Infect Dis.

[12] Abubakar I (2018). Prognostic value of interferon-γ release assays and tuberculin skin test in predicting the development of active tuberculosis (UK PREDICT TB): a prospective cohort study. Lancet Infect Dis.

[13] Trajman A, Steffen RE, Menzies D (2013). Interferon-gamma release assays versus tuberculin skin testing for the diagnosis of latent tuberculosis infection: an overview of the evidence. Pulm Med.

[14] World Health Organization (2022). Use of alternative interferon-gamma release assays for the diagnosis of TB infection: WHO policy statement, 2022.

[15] World Health Organization WHO global lists of high burden countries for tuberculosis (TB), TB/HIV and multidrug/rifampicin-resistant TB (MDR/RR-TB), 2021–2025: background document.

[16] World Health Organization (2022). Tuberculosis profile: Viet Nam.

[17] Hoa NB (2013). First national tuberculin survey in Viet Nam: characteristics and association with tuberculosis prevalence. Int J Tuberc Lung Dis.

[18] Fox GJ (2017). Latent tuberculous infection in household contacts of multidrug-resistant and newly diagnosed tuberculosis. Int J Tuberc Lung Dis.

[19] Nguyen BH (2010). National survey of tuberculosis prevalence in Viet Nam. Bull World Health Organ.

[20] Nguyen HV (2020). The second national tuberculosis prevalence survey in Vietnam. PLoS One.

[21] Chandrasekaran P (2018). Tuberculin skin test and QuantiFERON-Gold In Tube assay for diagnosis of latent TB infection among household contacts of pulmonary TB patients in high TB burden setting. PLoS One.

[22] Gao L (2015). Latent tuberculosis infection in rural China: baseline results of a population-based, multicentre, prospective cohort study. Lancet Infect Dis.

[23] Marks GB (2018). Prevalence of latent tuberculous infection among adults in the general population of Ca Mau, Viet Nam. Int J Tuberc Lung Dis.

[24] Herzmann C (2016). Risk for latent and active tuberculosis in Germany. Infection.

[25] Prakash Babu S (2023). Comparison of IGRA and TST in the diagnosis of latent tuberculosis among women of reproductive age in South India. Indian J Tuberc.

[26] Paradkar M (2020). Tuberculosis preventive treatment should be considered for all household contacts of pulmonary tuberculosis patients in India. PLoS One.

[27] Chauhan A (2023). The prevalence of tuberculosis infection in India: A systematic review and meta-analysis. Indian J Med Res.

[28] Lu P (2022). Selection of the cutoff value of the tuberculin skin test for diagnosing students who need preventive treatment: a school-based cross-sectional study. Front Cell Infect Microbiol.

[29] Villarino ME (1999). Comparable specificity of 2 commercial tuberculin reagents in persons at low risk for tuberculous infection. JAMA.

[30] Mulder C (2019). Tuberculin skin test reaction depends on type of purified protein derivative: implications for cut-off values. Int J Tuberc Lung Dis.

[31] Chumpa N (2022). Prevalence of latent tuberculosis infection among pre-clinical and clinical medical students using QuantiFERON-TB gold plus and tuberculin skin test at a teaching hospital in Thailand: a cross-sectional study. J Infect Public Health.

[32] Central TB Division Ministry of Health & Family Welfare, National TB Elimination Programme (2021). Guidelines for programmatic management of tuberculosis preventive treatment in India.

[33] Hamada Y (2021). Framework for the evaluation of new tests for tuberculosis infection. Eur Respir J.

[34] World Health Organization (2022). Module 3: diagnosis. Tests for tuberculosis infection.

[35] Aggerbeck H (2018). C-Tb skin test to diagnose *Mycobacterium tuberculosis* infection in children and HIV-infected adults: a phase 3 trial. PLoS One.

