# Supplementary material for: Tuberculin skin testing and QuantiFERON™-TB Gold Plus positivity among household contacts in Vietnam
Source: Public Health Action. 2023 Sep 21;13(3):83–9. doi: 10.5588/pha.23.0020 (PMC10446657; doi:10.5588/pha.23.0020)
Supplement: Supplementary file 1 [file iutld_pha_23.0020_supplementarydata1.pdf]

PHA 0020
http://dx.doi.org/10.5588/pha.23.0020

SUPPLEMENTARY DATA

Tuberculin skin testing and QuantiFERON-TB Gold Plus positivity among household contacts in Vietnam

Supplementary Figure S1. Quantitative QFT results for 2020 and 2021.

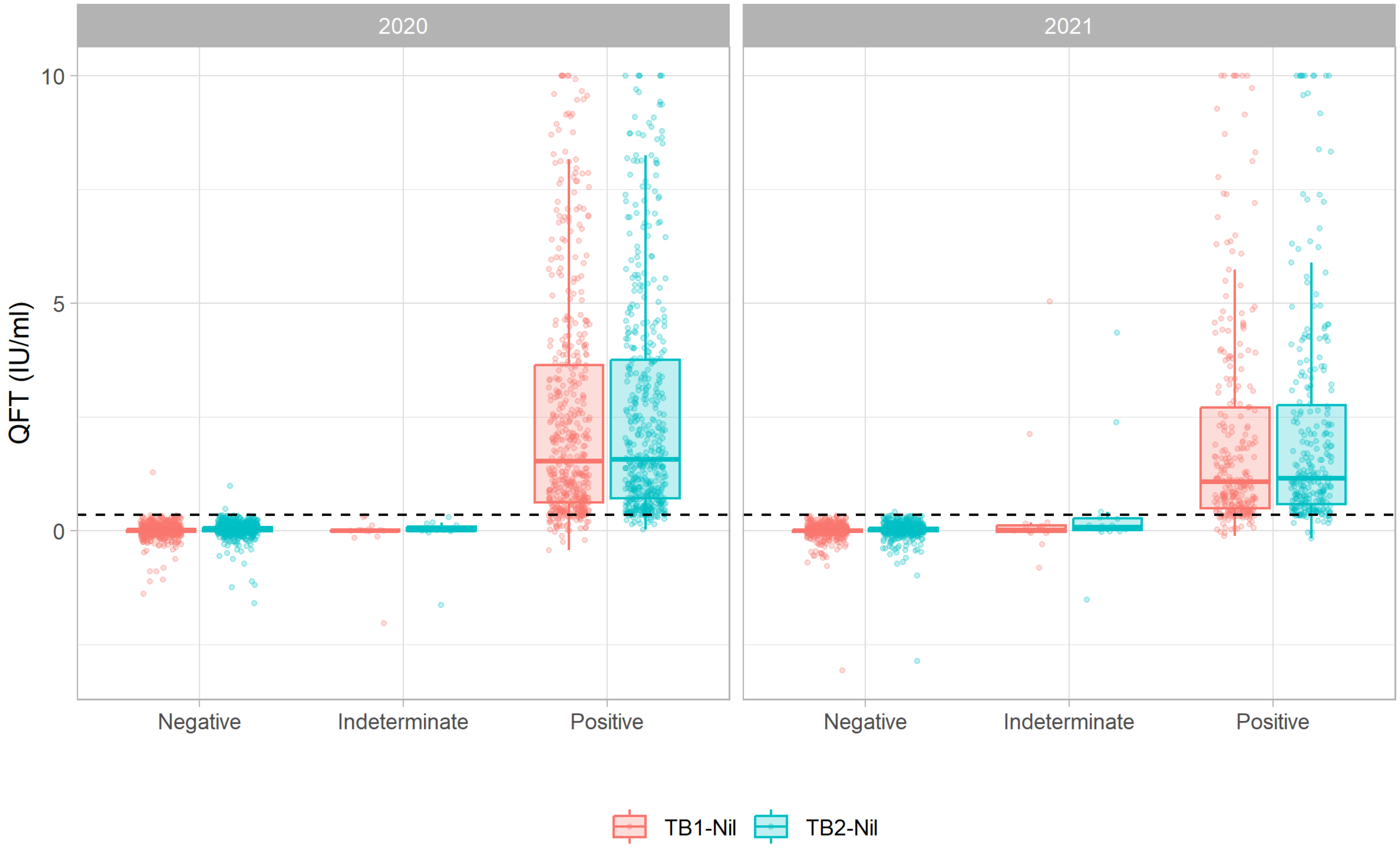

The dotted line is the threshold for QFT positivity ≥0.35 IU/ml. Quantitative QFT results are reported for 2020 (n = 1,344) and 2021 (n = 1,174). Positive QFT (TB1-Nil and TB2-Nil) results show a wide distribution with clustering close to the QFT positivity

threshold (0.2 – 0.5 IU/ml). IU = international units, IQR = interquartile range; QFT = QuantiFERON-TB Gold Plus, TB1 = primarily detects CD4 optimised antigens, TB2 = contains additional peptides optimised for stimulating CD4 and CD8 cells.

Supplementary Table S1: Results from univariable and multivariable analyses for QFT, TST ≥5mm and TST ≥10mm for 2020 and 2021.

	n (%) Positive	χ^2 P-value	QFT-Plus positivity	n (%) Positive	χ^2 P-value	TST ≥5mm aOR (95% CI)	n (%) Positive	χ^2 P-value	TST ≥10mm

			aOR (95% CI)						aOR (95% CI)
**2020 (n = 1,330)**	(n = 514)		(n=1,327)	(n = 498)		(n = 1,327)	(n = 174)		(n = 1,327)
Sex		0.890			0.345			0.683	
Female	325 (63.2%)		Reference	308 (61.8%)		Reference	108 (62.1%)		Reference
Male	189 (36.8%)		1.2 (0.9, 1.5)	190 (38.2%)		1.2 (0.9, 1.6)	66 (37.9%)		1.2 (0.8, 1.6)
Age Group		<0.001			<0.001			0.027	
0 to <10	15 (2.9%)		0.6 (0.3, 1.1)	14 (2.8%)		0.5 (0.3, 1.1)	4 (2.3%)		0.5 (0.2, 1.6)
10 to <20	33 (6.4%)		Reference	33 (6.6%)		Reference	11 (6.3%)		Reference
20 to <30	38 (7.4%)		1.3 (0.7, 2.2)	55 (11.0%)		2.2 (1.3, 3.9)	19 (10.9%)		1.9 (0.9, 4.3)
30 to <40	85 (16.6%)		2.0 (1.2, 3.3)	82 (16.5%)		1.9 (1.2, 3.2)	28 (16.1%)		1.7 (0.8, 3.5)

40 to <50	93 (18.2%)		2.5 (1.5, 4.0)	69 (13.9%)		1.7 (1.0, 2.8)	29 (16.7%)		1.7 (0.8, 3.6)
50 to <60	117 (22.9%)		2.6 (1.6, 4.2)	113 (22.7%)		2.5 (1.6, 4.1)	40 (23.0%)		2.0 (1.0, 4.2)
60 to <70	99 (19.3%)		3.1 (1.9, 5.0)	103 (20.7%)		3.5 (2.1,5.8)	36 (20.7%)		2.4 (1.2, 5.0)
70 and older	32 (6.3%)		3.0 (1.6, 5.6)	29 (5.8%)		2.8 (1.4, 5.3)	7 (4.0%)		1.3 (0.5, 3.5)
South region		<0.001			<0.001			0.167	
No	86 (16.7%)		Reference	50 (10.0%)		Reference	33 (19.0%)		Reference
Yes	428 (83.3%)		2.0 (1.5, 2.6)	448 (90.0%)		4.1 (2.9, 5.8)	141 (81.0%)		1.3 (0.9, 1.9)
**2021 (n = 1,158)**	(n = 279)		(n = 1,122)	(n = 305)		(n = 1,122)	(n = 129)		(n = 1,112)
Sex		0.697			0.011			0.283	
Female	173 (64.1%)		Reference	168 (56.9%)		Reference	74 (58.7%)		Reference

Male	97 (35.9%)		1.1 (0.8, 1.4)	127 (43.1%)		1.6 (1.2, 2.1)	52 (41.3%)		1.3 (0.9, 1.9)
Age Group		<0.001			0.001			0.138	
0 to <10	13 (4.7%)		0.6 (0.3, 1.2)	16 (5.3%)		0.7 (0.4, 1.4)	5 (3.9%)		0.5 (0.2,1.6)
10 to <20	30 (10.8%)		Reference	28 (9.2%)		Reference	12 (9.3%)		Reference
20 to <30	17 (6.1%)		0.9 (0.4, 1.7)	29 (9.5%)		2.0 (1.1, 3.8)	15 (11.6%)		2.1 (0.9, 4.8)
30 to <40	38 (13.6%)		1.0 (0.6, 1.7)	47 (15.5%)		1.5 (0.9, 2.7)	21 (16.3%)		1.4 (0.6, 2.9)
40 to <50	35 (12.5%)		1.3 (0.7, 2.3)	41 (13.5%)		1.8 (1.0, 3.1)	16 (12.4%)		1.4 (0.7, 3.2)
50 to <60	60 (21.5%)		1.4 (0.9, 2.4)	63 (20.7%)		1.8 (1.1, 3.1)	29 (22.5%)		1.7 (0.8, 3.5)
60 to <70	57 (20.4%)		2.4 (1.4, 4.0)	55 (18.1%)		2.9 (1.7, 5.0)	21 (16.3%)		2.0 (0.9, 4.3)
70 and older	29 (10.4%)		2.1 (1.2, 4.0)	25 (8.2%)		2.1 (1.1, 4.0)	10 (7.8%)		1.6 (0.7, 4.0)

South region	0.012		0.625		0.389	
No	66 (23.7%)	Reference	87 (28.5%)	Reference	34 (26.4%)	Reference
Yes	213 (76.3%)	1.5 (1.1, 2.1)	218 (71.5%)	0.9 (0.7, 1.2)	95 (73.6%)	1.1 (0.7, 1.7)

Symptoms (cough, fever, weight loss, and night sweats) were included in the multivariable model but did not increase or decrease the odds for test positivity, with one exception: in 2020, weight loss increased odds for QFT positivity, aOR 1.7, 95% CI 1.0, 2.9). χ^2 = Chi-square; aOR = adjusted odds ratio; CI = confidence interval; TST= tuberculin skin testing; QFT = QuantiFERON-TB Gold Plus.

Supplementary Table S2. Pairwise comparisons of age-specific positivity rates for QFT, TST≥5mm and TST≥10mm for 2020 and 2021.

	Year	Age-specific positivity rate 1	Age-specific positivity rate 2	Mean difference	P-value
	2020	QFT+/TST+	QFT–/TST+	3.7	0.148
TST≥5mm	2020	QFT+/TST+	QFT+/TST–	3.2	0.250
	2020	QFT–/TST+	QFT+/TST–	-0.4	0.859
	2021	QFT+/TST+	QFT–/TST+	-3.2	0.040
	2021	QFT+/TST+	QFT+/TST–	-0.9	0.496
	2021	QFT–/TST+	QFT+/TST–	2.3	0.261
	2020	QFT+/TST+	QFT–/TST+	5.4	<0.001
TST≥10mm	2020	QFT+/TST+	QFT+/TST–	-18.8	<0.001
	2020	QFT–/TST+	QFT+/TST–	-24.3	<0.001
	2021	QFT+/TST+	QFT–/TST+	1.9	0.020
	2021	QFT+/TST+	QFT+/TST–	-10.9	0.003
	2021	QFT–/TST+	QFT+/TST–	-12.8	0.002

Table S2 Upper (TST≥5mm) and Lower (TST≥10mm) panels align with Figure 2 Middle and Lower panels. **Lower (**TST≥10mm**).** For both 2020 and 2021, the paired [QFT–/TST+, QFT+/TST–] age-specific positivity rates for TST≥10mm are statistically different (2020 mean difference = –24.3, P <0.001; 2021 mean difference = –12.8, P = 0.002). **Upper (**TST≥5mm**).** For 2020 and 2021, paired [QFT–/TST+, QFT+/TST–] age-specific positivity rates for TST≥5mm are not statistically different (2020 mean difference = –0.4, P = 0.859; 2021 mean difference = 2.3, P = 0.261). The Bonferroni correction adjusted for six independent hypothesis tests in each panel; to maintain an overall significance level of 0.05, the alpha level was adjusted to 0.008 (0.05 divided by 6) for each test. QFT = QuantiFERON TB Gold-Plus; TST = tuberculin skin testing, P = probability associated with a t-test
